# Dr. Otis Avery

**Published:** 1904-04

**Authors:** 


### Obituary.

### DR. OTIS AVERY.[1]

DIED, Monday, February 22, 1904, Dr. Otis Avery.

Dr. Avery was born in Bridgewater, Oneida County, N. Y., August 19, 1808. At the time of his death he had nearly completed his ninety-sixth year, and continued to practice his profession until his ninety-fourth year. At that time he was regarded as the oldest living dental practitioner in the United States, and this was undoubtedly true, certainly as far as practice was concerned.

Dr. Avery was one of the remarkable men of the last century. He was a many-sided man, and this versatility, in some degree, prevented his obtaining a deserved national reputation in the profession of his choice. A writer briefly sums up his life-work in the order of acquirement: "Silversmith, watchmaker, dental surgeon, inventor, statesman, philosopher, judge, and one of the most remarkable men of genius of the century."

"The paternal grandfather of the deceased was born at Groton, Conn., and served all through the Revolutionary War, after which conflict he engaged in teaching, and lived to be nearly a century old. The father of the subject of this sketch was also born at Groton, but removed to Oneida, where he was identified with its very earliest settlement. He was a silversmith and watchmaker, and taught his son the trade.

"At the age of fifteen our subject left home and worked for awhile as a journeyman at Waterville, N. Y., and three years afterwards removed to Cochecton, where he served as clerk in a store for his brother John and afterwards opened there a jewelry and repair shop. While at that place he became a captain of a military company. In 1827 he came to Bethany, where he established a shop, and from that place removed to New Berlin, N. Y., where he continued his vocation. While there he decided to become a dentist, and, going to New York City, passed two years with Dr. D. C.

[1] The INTERNATIONAL is indebted to Judge George S. Purdy, of Honesdale, for valuable papers connected with Dr. Avery's life.

Ambler, a prominent dentist, who on December 6, 1833, gave to Dr. Avery probably the first certificate ever issued to a dentist in this country, as at that time there were no dental schools or dental supply houses in America. During the next four years Dr. Avery practised his profession over a large area of country, one of his fields being between Honesdale and Ithaca, N. Y. In 1839 he located at Bethany, where for ten years he spent the summer, going to Columbia, S. C., every winter. Subsequently he opened an office in New York City, but finally gave up the practice there and in 1850 settled permanently at Honesdale. His skill and prominence in the profession were largely the result of his genius, self-education, and constant research. Besides pursuing his profession he gave attention to invention, and the year he settled here patented a sewing-machine, which he sold to a company and as its agent visited Europe and sold the patent to parties in London and also to Emperor Louis Napoleon in behalf of the French government. In 1851 he received from Prince Albert, President of the Royal Commission having in charge an exhibition of the works of industry of all nations, a medal for superior dentistry, and a similar medal the following year from the agricultural society of the State of New York."

In 1850 Dr. Avery removed to Honesdale, Pa. In 1855 he was sent to the State Legislature, where he served one term. He was appointed one of the associate judges of Wayne County in 1871, and in 1877 was re-elected by an overwhelming vote; but at the end of his second term he declined further service. It is probably the first and only instance on record of a judge descending voluntarily from the bench to practice dentistry, or of a dentist being elevated to that position.

An interesting fact in Dr. Avery's life was his riding on the "Stourbridge Lion," the first locomotive seen in this country. This was on August 8, 1829. "We," he writes, "were determined to see what we could of the thing, and went up to where it stood. The man in charge was just emptying the fire from under the boiler and quenching it with water. We stepped upon the platform and I asked him if he could not start it so that we might see how it worked. He let on the steam and ran it about the middle of the river. I told him that would do, and he reversed it and ran it onto the land." It is difficult to realize the fact that one lifetime has witnessed the development, from that single imported locomotive, to the great railroad interests dominating this country. It vividly brings to mind the progress made in the nineteenth century.

In one of the last letters of Dr. Avery written to the editor of the INTERNATIONAL, August 18, 1903, he stated that he was interested in an editorial article entitled "Avoid the Road to the Poor-House," and further wrote: "Your valuable suggestions ought to be put in operation, for it is a conceded fact that ten or a dozen years in dental occupation unfit a man for any other business, and there should be some means provided to protect him from the sharpers, especially that abomination the 'pious fraud.'"

No one could fail to appreciate the high character of this man when brought in personal contact with him. He lived quietly and unostentatiously, yet, while largely isolated from the active work of his profession, he contributed much to maintain its dignified character and honorable position among the callings of men. A writer sums up the character of Judge Avery briefly, but very truthfully, as the present writer of this sketch knew him.

"He was always conspicuous for independent thought, positive convictions, unflinching courage, and spotless integrity; and those characteristics were abundantly displayed in the discharge of his official duties. He was not content to accept the current tradition relative to the position of a lay judge, and to pose as a mere judicial figure-head. On the contrary, his official career was marked by the active, intelligent, and conscientious discharge of its duties, and his influence was largely felt in the administration of justice."

At his death he was connected with the First Presbyterian Church at Honesdale.

He was twice married, first, April 19, 1829, to Louisa Hoel, of Bethany. She died in 1853. He subsequently married, in 1855, Mary Agnes Addoms, of New York, daughter of Richard Clark, a former merchant of that city.

The services were held at his home, Park Street, Honesdale, the final interment taking place at New Dorp, on Staten Island.

